# Transmission of fungi and protozoa under grazing conditions from lactating yaks to sucking yak calves in early life

**DOI:** 10.1007/s00253-023-12616-y

**Published:** 2023-06-21

**Authors:** Wei Guo, Tingmei Liu, André Luis Alves Neves, Ruijun Long, Allan Degen, Mi Zhou, Xiang Chen

**Affiliations:** 1 Key Laboratory of Animal Genetics, Breeding and Reproduction in the Plateau Mountainous Region, Ministry of Education, Guizhou University, Guiyang, 550025 China; 2 State Key Laboratory of Grassland Agro-Ecosystems, International Centre of Tibetan Plateau Ecosystem Management, School of Life Sciences, Lanzhou University, Lanzhou, 730000 China; 3 Department of Agricultural, Food and Nutritional Science, University of Alberta, EdmontonAlberta, AB T6G 2P5 Canada; 4 Department of Veterinary and Animal Sciences, Faculty of Health and Medical Sciences, University of Copenhagen, Grønnegårdsvej 3, 1870 Frederiksberg C, Denmark; 5 Desert Animal Adaptations and Husbandry, Wyler Department of Dryland Agriculture, Blaustein Institutes for Desert Research, Ben-Gurion University of the Negev, 8410500 Beer Sheva, Israel

**Keywords:** Eukaryotic microbes, Dam-to-infant transmission, Rumen microbial development, Maternal contribution

## Abstract

**Abstract:**

Microbiota from mothers is an essential source of microbes in early-life rumen microbiota, but the contribution of microbiota from different maternal sites to the rumen microbiota establishment in neonates needs more data. To fill this gap, we collected samples from the mouth, teat skin, and rumen of lactating yaks and from the rumen of sucking calves concomitantly on seven occasions between days 7 and 180 after birth under grazing conditions. We observed that the eukaryotic communities clustered based on sample sites, except for the protozoal community in the teat skin, with negative correlations between fungal and protozoal diversities in the rumen of calves. Furthermore, fungi in the dam’s mouth, which is the greatest source of the calf’s rumen fungi, accounted for only 0.1%, and the contribution of the dam’s rumen to the calf’s rumen fungi decreased with age and even disappeared after day 60. In contrast, the average contribution of the dam’s rumen protozoa to the calf’s rumen protozoa was 3.7%, and the contributions from the dam’s teat skin (from 0.7 to 2.7%) and mouth (from 0.4 to 3.3%) increased with age. Thus, the divergence in dam-to-calf transmissibility between fungi and protozoa indicates that the foundation of these eukaryotic communities is shaped by different rules. This study provides the first measurements of the maternal contribution to the fungal and protozoal establishment in the rumen of sucking and grazing yak calves in early life, which could be beneficial for future microbiota manipulation in neonatal ruminants.

**Key points:**

*• Dam to calf transfer of rumen eukaryotes occurs from multiple body sites.*

*• A minor proportion of rumen fungi in calves originated from maternal sites.*

*• The inter-generation transmission between rumen fungi and protozoa differs.*

**Supplementary Information:**

The online version contains supplementary material available at 10.1007/s00253-023-12616-y.

## Introduction

Microbial colonization and development of newborn ruminants begin at birth and continue thereafter (Wang et al. 2019). Microbes are crucial not only for the health of the offspring but also for the long-term health of the host, including the activation and development of the immune system, development of the nervous system, and digestion of food (Yao et al. 2021). Emerging evidence has demonstrated that gut microbial colonization of neonatal ruminants could regulate rumen papillae morphology and growth of the rumen epithelium (Lin et al. 2019; Malmuthuge et al. 2019a, b) and thus affect the health and phenotype of the host in adulthood (Malmuthuge et al. 2019a, b; Fan et al. 2021). Previous studies reported that early rumen microbiota colonization was characterized by rapid and dynamic variations in composition and function (Friedman et al. 2017; Guo et al. 2022). Microbiota was present in the rumen of dairy calves as early as 20 min post-partum (Guzman et al. 2015) and in the rumen of lambs by 1–3 days post-partum (Fonty et al. 1987; Morvan et al. 1994). It was proposed that the rumen microbiota of goat kids can alter rumen function (Lv et al. 2019) and that rumen microbes in lambs can stimulate the rumen morphological development through the production of fermentation end-products, such as volatile fatty acids (Li et al. 2022). In addition, the rumen development of neonatal calves could be stimulated by the rumen microbiome via miRNA expressions (Malmuthuge et al. 2019a, b). However, most studies to date have focused on the prokaryotic organisms (bacteria and archaea) and have basically ignored eukaryotic microbes (fungi and protozoa), although they play a pivotal role in the health of the host and digestion of fibers (Laforest-Lapointe and Arrieta 2018; Mizrahi and Jami 2018).

Studies in ruminants demonstrated that the establishment of gut microbiota in early life is influenced by various factors associated with the maternal microbiota (Guo et al. 2020a, b), feeding modes (Bi et al. 2019), rearing regimes (Belanche et al. 2019; Palma-Hidalgo et al. 2021), and environmental factors (Virgínio Júnior and Bittar 2021). The maternal vaginal and milk microbiota were reported to be the major sources of gut microbiota of infants, including goat kids, in the first 56 days of life (Guo et al. 2020a, b). In addition, feeding modes affect mother-to-infant transmission, where gut microbiota of suckled lambs was derived mainly from maternal teats, and those of bottle-fed lambs were derived mainly from the maternal birth canal (Bi et al. 2019). Maternal fecal microbiota was reported to be the main driver of fecal microbiota of yak and cattle calves (Zhang et al. 2022). However, only a few studies have assessed the contributions of maternal microbiota to the colonization and succession of the rumen microbiota of yak calves under grazing regimes (Guo et al. 2022). Multiple maternal sites (rumen, milk, teat skin, and mouth) contribute to the calves’ rumen bacteria and archaea development, with maternal rumen microbiota accounting for the largest proportion of colonizing microorganisms (Guo et al. 2022). Yaks (*Bos grunniens*) have various anatomical and physiological traits which enable them to cope with the harsh environment on the Qinghai-Tibetan Plateau and only graze natural pasture without supplements all year (Qiu et al. 2012). Furthermore, the rumen microbiome composition of yaks was found to be distinct from low-altitude cattle (Zhang et al. 2016) and exhibited unique developmental patterns (Guo et al. 2020a, b). Therefore, the current knowledge regarding the early-life rumen microbiota in captive ruminants cannot be applied to grazing yaks, as the feeding systems affect the early-life rumen microbial profiles (Jiao et al. 2015).

In addition, the sources of the yak calves’ rumen fungi and protozoa and the relationship between them during the developmental processes, which may contribute to the maturation of rumen microbes, are uncertain. To fill this gap, we examined the colonization process of rumen fungi and protozoa in yak calves. This study could provide insights into early acquisition of rumen fungi and protozoa, subsequent development, and potential strategies to manipulate rumen development. We hypothesized that the dam is an important source for the succession of rumen fungi and protozoa of grazing yak calves in early life because they sucked milk and grazed with the dams, and that relationships existed between rumen fungi and protozoa in yak calves during this period. To test this hypothesis, rumen fluid from yak calves sucking milk and grazing with their dams and samples from the mouth, teat skin, and rumen of dams were collected concomitantly on seven occasions from day 7 to day 180 of life. This enabled us to determine the contribution of different maternal body sites to the establishment and development of rumen fungi and protozoa in the yak calves.

## Materials and methods

### Animals and collection of samples

**Fig. 1:**
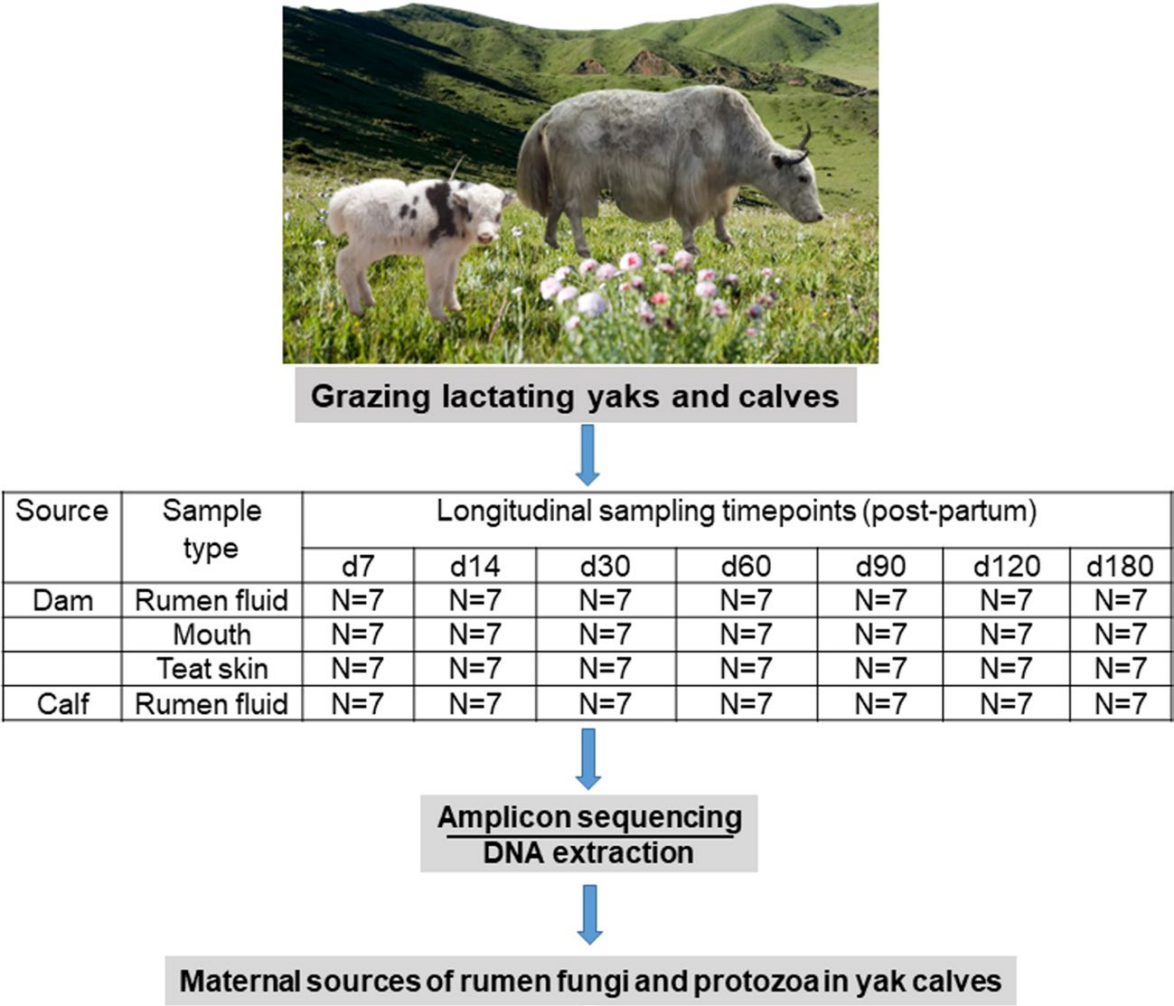
The experimental design depicting collection of samples and data analysis. Rumen, mouth, and teat skin samples of dams and rumen samples of calves were collected from 7 dam-calf pairs from day 7 to day 180 post-partum

Seven lactating yak cows (naks; 5–6 years old; 230 ± 16 kg) giving birth to male calves (15 ± 3 kg) between April 7 and 10, 2017, were used in this study. The calves sucked milk and grazed with the naks during the day in an alpine meadow on the Qinghai-Tibetan Plateau (Altitude: 3154 m; 37° 12.4′ N, 102° 51.7′ E). The annual air temperature ranges from − 8 to 4 °C. The naks were corralled and separated from the calves overnight. The flowchart for this study is presented in Fig. 1. The protocol used for collection of samples was described previously (Guo et al. 2022). In brief, rumen fluid samples were collected before morning grazing from the naks and calves on days 7, 14, 30, 60, 90, 120, and 180 post-parturition. A flexible oral stomach tube (Anscitech Co., Ltd. Wuhan, China) connected to a rumen pump was used. In addition, mouth and teat skin samples were collected from the lactating naks concomitantly with the rumen fluid samples following the protocols described by Ferretti et al. (2018). All samples were snap-frozen in liquid nitrogen immediately and stored at − 80 °C for DNA extraction and microbial characterization.

### DNA extraction and amplicon sequencing

The skin and mouth swabs were eluted in 500 μl of saline, vortexed for 2 min, and then centrifuged at 1000 g for 5 min. DNA extraction used PowerSoil DNA Isolation Kits (MoBio Laboratories Carlsbad, CA, USA) according to the manufacturer’s instructions. The quality and quantity of extracted DNA were evaluated using the NanoDrop 2000 spectrophotometer (Thermo Fisher Scientific, Waltham, MA, USA) and agarose gel electrophoresis, respectively. To determine the eukaryotic profiles, the protozoal 18S rRNA was amplified using the primer set Reg841F (5′-GACTAGGGATTGGAGTGG-3′) and Reg1302R (5′-AATTGCAAAGATCTATCCC-3′), and fungal sequences were amplified with the primers targeting the ITS1 region (MN100F-TCCTACCCTTTGTGAATTTG, MNGM2-CTGCGTTCTTCATCGTTGCG) (Guo et al. 2020a, b). The amplicon libraries were sequenced using the 2 × 300 paired-end protocol on the Illumina MiSeq platform (Majorbio Company, Shanghai, China). The sequencing data of this study are available at the NCBI Sequence Read Archive under accession number PRJNA838115.

### Bioinformatics analysis

Sequencing data were processed with QIIME2-version 2020.2 (Bolyen et al. 2019). Briefly, quality control, denoising, combined paired-ends, and removal of chimeras were conducted using the DADA2 algorithm of QIIME2 (Callahan et al. 2016). High-quality reads were classified into amplicon sequence variations (ASV) based on 99% sequence identity. ASVs were classified against the UNITE database for fungi and SILVA 132 database for protozoa using a Naïve Bayes classifier implemented in the *q2-feature-classifier* plugin. A phylogenetic tree was constructed for diversity analyses using the *align-to-tree-mafft-fasttree* command (Katoh and Standley 2013; Price et al. 2010). Before calculating the alpha (Shannon and Chao1 indices) and beta diversities (Bray–Curtis distances), feature tables were rarefied to 8769 for fungi and 1822 for protozoa sequences per sample, and the diversity metrics were computed using the *core-metrics-phylogenetic* command. To determine the proportion of rumen fungi and protozoa of yak calves that originated from their dams, SourceTracker2 (Knights et al. 2011), a Bayesian community-level microbial source-tracking tool, was employed. In this analysis, SourceTracker2 was run with default parameters on the non-rarefied data; rumen samples from yak calves were set as “sink,” and samples from naks (rumen fluid, mouth, and teat skin) were set as “source.”

### Statistical analysis

Statistical analysis was conducted in *RStudio* (v 3.5.3) (Allaire 2012). The Kruskal–Wallis test assessed changes in the alpha diversity indices and abundances of microbial taxa among different maternal sites and across calf ages, and significant differences among two sites within each age group were separated using Dunn’s test following a false discovery rate (FDR) correction for multiple comparisons by the Benjamini–Hochberg method (Ferreira and Zwinderman 2006). Adjusted *P* values < 0.05 were considered statistically significant. Ordination used non-metric multidimensional scaling (NMDS) based on Bray–Curtis dissimilarity to compare the overall dissimilarity of eukaryotic communities among different sampling sites and across ages, the significant value was determined with ANOSIM implemented in the “vegan” package (Dixon 2003). The Spearman’s correlation analysis tested the relationships between rumen fungi and protozoa in yak calves during the developmental progress, and significance was accepted at *P* < 0.05. Taxa at phylum and genus levels that were present in more than 50% of the individuals at each site within each age group (relative abundance > 1%) were defined as detected taxa for downstream analysis (Guo et al. 2022).

## Results

### Microbial diversity of fungal community in dams and calves

We applied amplicon sequencing to all 245 samples, including 49 from calves and 196 from dams (Fig. 1). Fungi and protozoa were both detected in the rumen of calves at 30 days of age, and thus, further analysis continued from 30 days of age with only 140 samples in the following sections. From the cohort, high-quality sequence data were obtained for 76 fungi and 112 protozoa samples (Table 1).

**Table 1:** Sample information and sequencing statistics obtained after using the DADA2 algorithm implemented in QIIME2

Microbial group	Age group	Source	Site	Number of samples	Number of sequences	Frequency^a^	Number of ASVs^b^
Fungi	d30	Calf	Rumen fluid	5	63,932 ± 5414	50,368 ± 6380	27 ± 4
		Dam	Rumen fluid	4	64,621 ± 5753	52,149 ± 3961	48 ± 9
			Mouth	6	50,169 ± 9858	34,297 ± 6406	43 ± 9
	d60	Calf	Rumen fluid	5	63,156 ± 3992	45,011 ± 6740	49 ± 8
		Dam	Rumen fluid	6	68,114 ± 3421	57,191 ± 3510	40 ± 6
			Mouth	5	53,007 ± 8808	37,402 ± 6488	63 ± 6
	d90	Calf	Rumen fluid	5	65,614 ± 6234	47,124 ± 3986	51 ± 8
		Dam	Rumen fluid	6	60,305 ± 4905	50,926 ± 4721	38 ± 8
			Mouth	5	67,872 ± 2764	53,040 ± 3572	44 ± 10
	d120	Calf	Rumen fluid	6	67,611 ± 3026	50,770 ± 3340	46 ± 8
		Dam	Rumen fluid	5	71,322 ± 1023	63,683 ± 925	35 ± 9
			Mouth	5	57,171 ± 5681	43,937 ± 4290	48 ± 12
	d180	Calf	Rumen fluid	5	69,635 ± 2058	33,503 ± 10,770	41 ± 9
		Dam	Rumen fluid	4	68,082 ± 3561	59,876 ± 3144	44 ± 16
			Mouth	4	54,586 ± 6769	32,523 ± 5566	60 ± 8
Protozoa	d30	Calf	Rumen fluid	6	22,031 ± 2836	14,055 ± 2268	22 ± 2
		Dam	Rumen fluid	5	18,513 ± 1761	6504 ± 749	42 ± 4
			Mouth	5	16,492 ± 1899	9557 ± 740	38 ± 4
			Skin	6	18,845 ± 2053	9986 ± 866	27 ± 4
	d60	Calf	Rumen fluid	5	18,361 ± 832	10,826 ± 1209	10 ± 2
		Dam	Rumen fluid	5	20,774 ± 2288	7964 ± 841	34 ± 4
			Mouth	5	21,154 ± 1563	11,153 ± 707	56 ± 4
			Skin	6	15,125 ± 1833	8344 ± 1048	30 ± 7
	d90	Calf	Rumen fluid	6	16,334 ± 1268	6163 ± 1080	18 ± 2
		Dam	Rumen fluid	6	17,076 ± 1854	7122 ± 879	32 ± 4
			Mouth	5	19,125 ± 3408	10,507 ± 3319	51 ± 4
			Skin	6	18,031 ± 1852	10,149 ± 1010	16 ± 2
	d120	Calf	Rumen fluid	7	17,212 ± 1842	6573 ± 930	21 ± 4
		Dam	Rumen fluid	6	22,689 ± 1938	7780 ± 2281	32 ± 2
			Mouth	5	17,576 ± 1962	9808 ± 1296	58 ± 5
			Skin	7	18,493 ± 2318	10,468 ± 1421	27 ± 5
	d180	Calf	Rumen fluid	6	20,446 ± 2397	5709 ± 665	32 ± 3
		Dam	Rumen fluid	5	18,601 ± 2314	5455 ± 1352	45 ± 7
			Mouth	5	19,790 ± 1615	12,902 ± 1074	64 ± 11
			Skin	5	15,933 ± 2490	8394 ± 1929	32 ± 8

^a^Frequency, the average number of high-quality reads per sample after DADA2 sequence quality control

^b^*ASVs*, amplicon sequence variations

**Fig. 2:**
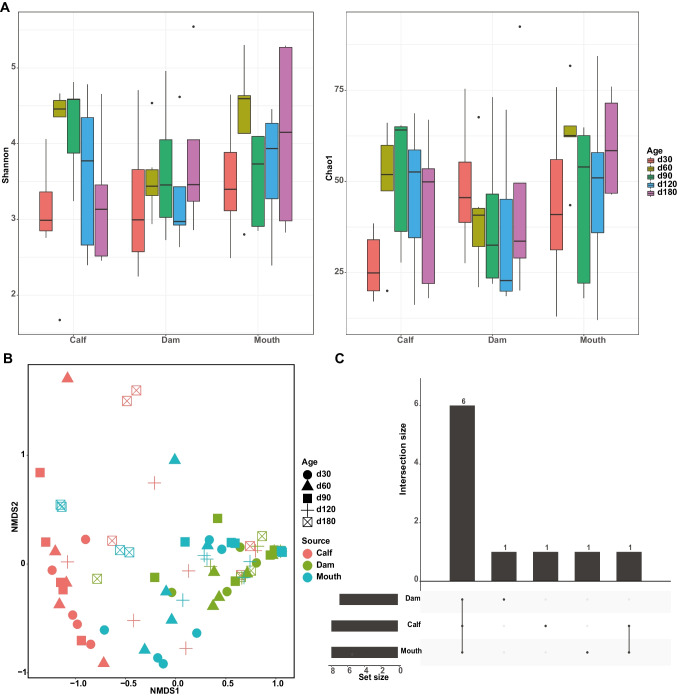
Fungal communities in the maternal and calf samples are diverse and affected by sampling site and time. **A** Alpha diversity measured by Shannon and Chao1 indices at different sample types across ages. **B** Non-metric multi-dimensional scaling plot based on Bray–Curtis distances. **C** UpSet plot showing the number of differential and shared fungal genera among different sample types

Amplicon sequencing of the retained fungi samples (dam rumen: *n* = 25; mouth: *n* = 25; calf rumen: *n* = 26) generated a total of 3,607,668 high-quality reads with a median of 47,469 (SEM = 1652), representing 3403 amplicon sequence variants (ASVs; 45 ± 2) (Table 1). Fungi were not detected in the skin samples. The average Good’s coverage estimation for all individuals was 99.8% (SEM = 0.001), indicating that the eukaryotic organisms present in the samples were completely sequenced in this study. The Shannon and Chao1 indices did not differ among sites within each age group (Supplemental Fig. S1A), and the Shannon and Chao1 indices in the rumen of calves were higher at day 60 than day 30 (*P* < 0.05) and not different between any other time points (*P* > 0.05, Fig. 2A), while those of dam rumen and mouth samples fluctuated over time (Fig. 2A). Notably, an NMDS ordination plot based on Bray–Curtis distance revealed that differences in fungal community varied not only according to sample types (ANOSIM R = 0.3, *P* < 0.01, Fig. 2B) but also across ages (ANOSIM R = 0.05, *P* < 0.05, Fig. 2B). The shared and unique taxa across the three sample sites are presented in Fig. 2C. The results showed that the sample-specific core genera were relatively low, and the shared genera were abundant (Fig. 2C).

### Microbial diversity of protozoal community in dams and calves

**Fig. 3:**
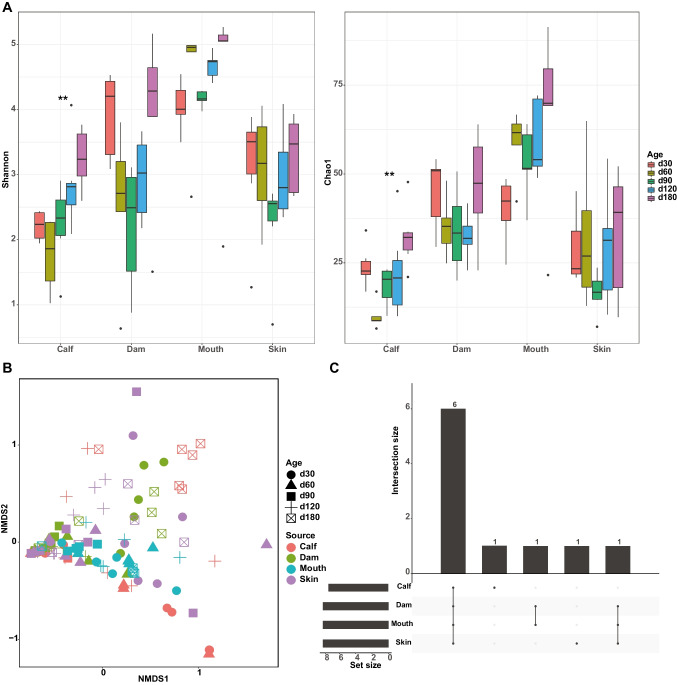
Protozoal communities in the maternal and calf samples are diverse and affected by sampling site and time. **A** Alpha diversity measured by Shannon and Chao1 indices at different sample types across ages. **B** Non-metric multi-dimensional scaling plot based on Bray–Curtis distances. **C** UpSet plot showing the number of differential and shared protozoal genera among different sample types. *P < 0.05, **P < 0.01

For protozoal communities, 112 samples (calf rumen: *n* = 30; dam rumen: *n* = 27; mouth: *n* = 25; teat skin: *n* = 30) were amplicon sequenced, yielding a total of 1,000,537 high-quality sequences, with a mean read count of 8933 (SEM = 365), represented by 3739 ASVs (33 ± 2 per sample) (Table 1). A decreased protozoal alpha diversity, assessed by the Shannon and Chao1 indices, was observed for calves from day 30 to day 60 followed by a significant increase (*P* < 0.05, Fig. 3A). Both Shannon and Chao1 indices of calf rumen protozoa were lower than in the dam’s mouth at all time points, except at day 180 (*P* < 0.05, Supplemental Fig. S1B). The alpha diversity indices (Shannon and Chao1) of mouth and skin samples varied greatly during this period but did not reach significance (*P* > 0.05, Fig. 3A). Differences in the protozoal community between calf and maternal samples were visualized by multi-dimensional scaling (Fig. 3B) and tested by ANOSIM. The difference in protozoal composition among the samples was explained by age (ANOSIM R = 0.16, *P* < 0.001, Fig. 3B), while the sample site explained a small part of the variation in beta diversity (ANOSIM R = 0.06, *P* < 0.01, Fig. 3B). The UpSet plot showed that only calf and skin had site-unique taxa (*Haptoria* for calf and *Tracheophyta* for skin), while the most common genera (*Dasytricha*, *Entodinium*, *Trichostomatia*, *Polyplastron*, uncultured *Trichostomatia*, and *Ophryoscolex*) were present in all four sites (Fig. 3C).

### Composition of fungi in dams and calves

**Fig. 4:**
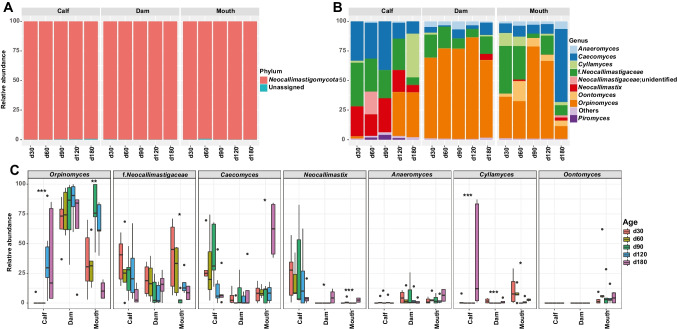
Different fungal communities in the maternal and calf samples. Relative abundances of fungal phyla (**A**) and genera (**B**) at different sites across ages. **C** Comparison of the relative abundances of the main fungi at different sites across ages at the genus level. *P < 0.05, **P < 0.01, ***P < 0.001

One phylum and nine genera of fungi were identified, based on the selection criteria stated in the materials and methods. *Neocallimastigomycota* was the dominant phyla at all sites at all ages (Fig. 4A; Supplemental Table S1). *Caecomyces* (25.3% ± 0.05), f.*Neocallimastigaceae* (24.4% ± 0.04), *Neocallimastix* (19.4% ± 0.05), and *Orpinomyces* (16.2% ± 0.06) were the predominant fungal genera in the rumen of calves, and their relative abundances fluctuated with age (Fig. 4B; Supplemental Table S2). Specifically, the relative abundance of *Orpinomyces* increased with age from 1.9 to 37.8% (*P* < 0.05), while the remaining dominant genera decreased from day 30 to day 180, although there was a fluctuation at day 90 (*P* < 0.05, Fig. 4B; Supplemental Table S2). Similarly, the dam’s rumen was dominated by *Orpinomyces* (75.2% ± 0.05), *Neocallimastigaceae* (13.4% ± 0.03), *Caecomyces* (5.1% ± 0.02), and *Anaeromyces* (4.1% ± 0.01) (Fig. 4B), and the relative abundance of *Anaeromyces* decreased with age (*P* < 0.05, Supplemental Table S2). The predominant fungal genera in the mouth were *Orpinomyces* (45.4% ± 0.06), *Neocallimastigaceae* (20.7% ± 0.04), *Caecomyces* (16.8% ± 0.05), *Oontomyces* (7.1% ± 0.03), and *Anaeromyces* (3.3% ± 0.01) (Fig. 4B; Supplemental Table S2). The relative abundances of *Anaeromyces* (from 1.8 to 6.5%) and *Caecomyces* (from 7.6 to 61.2%) remained stable from day 30 to day 120 but increased significantly from day 120 to day 180, whereas *Cyllamyces* decreased from day 30 to day 180 (from 10.9% to 0) (*P* < 0.05, Fig. 4B).

To further assess the fungi development in the rumen of calves in detail, we compared the differences of fungal communities among calf’s rumen, and dam’s mouth and rumen. Compared with the dam’s mouth and rumen, calves’ rumen had consistently lower abundances of *Anaeromyces* (*P* < 0.05, Fig. 4C), but higher *Caecomyces* and *Neocallimastix*, especially before day 180 (*P* < 0.05, Fig. 4C). The relative abundance of *Orpinomyces* in the dam’s rumen was higher than in the calf’s rumen and mouth (*P* < 0.05, Fig. 4C). *Oontomyces*, which dominated in the mouth, was not detected in the rumen of calves and dams (Fig. 4C).

### Composition of protozoa in dams and calves

**Fig. 5:**
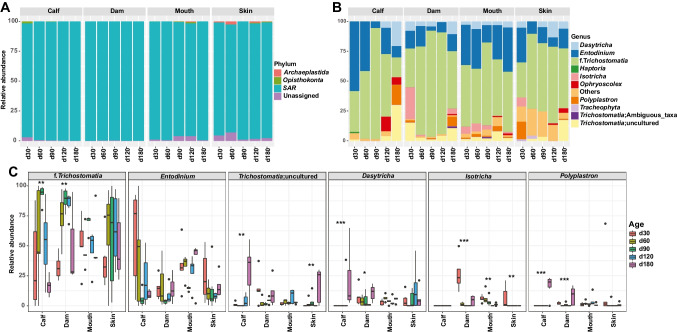
Different protozoal communities in the maternal and calf samples. Relative abundances of protozoal phyla (**A**) and genera (**B**) at different sites across ages. **C** Comparison of the relative abundances of the main protozoa at different sites across ages at the genus level. *P < 0.05, **P < 0.01, ***P < 0.001

Four protozoa phyla were identified according to the above-mentioned criteria, with *Stramenopila*-*Alveolata*-*Rhizaria* (*SAR*) being the most abundant (97.9% ± 0.01) in all samples, while *Archaeplastida* was detected only in the mouth (0.2% ± 0.001) and skin (1.1% ± 0.01) (Fig. 5A; Supplemental Table S1). *Opisthokonta*, which was detected in the rumen of calves (d30) and the dam’s mouth and skin, was not detected in the dam’s rumen (Fig. 5A). At the genus level, *Trichostomatia* (calf vs. dam; 50.5% ± 0.07 vs. 67.3% ± 0.05), *Entodinium* (26.8% ± 0.06 vs. 11.2% ± 0.02), and uncultured *Trichostomatia* (7.2% ± 0.03 vs. 6.6% ± 0.02) were prevalent in the rumen of both calves and dams, and their relative abundances varied with age (Fig. 5B; Supplemental Table S2). For example, the relative abundance of uncultured *Trichostomatia* increased from 1.2 to 30.1% in the rumen of calves but decreased from 37.3 to 2.6% in the rumen of dams (Supplemental Table S2). The dynamic changes in relative abundance of *Trichostomatia* in the rumen of calves and dams were similar, namely, an increase from day 30 to day 90 and then a decrease afterward (Supplemental Table S2). Notably, *Dasytricha* (5.0% ± 0.03) was not detected in the rumen of calves at days 30, 60, and 90, whereas it dominated at days 120 and 180 (Fig. 5B; Supplemental Table S2). *Isotricha* (6.2% ± 0.02) was dominant in the rumen of dams but was not detected in the rumen of calves (Fig. 5B; Supplemental Table S2).

In the mouth samples, *Trichostomatia* (52.9% ± 0.04), *Entodinium* (29.4% ± 0.03), *Dasytricha* (4.2% ± 0.01), and uncultured *Trichostomatia* (3.5% ± 0.01) were the predominant genera (Fig. 5B; Supplemental Table S2). The relative abundance of *Isotricha* decreased from 5.9% to 0 with age (*P* < 0.05, Fig. 5C). For teat skin, *Trichostomatia* (54.7% ± 0.06), *Entodinium* (14.3% ± 0.03), *Dasytricha* (6.0% ± 0.02), and uncultured *Trichostomatia* (4.0% ± 0.02) were the predominant genera (Fig. 5B; Supplemental Table S2). Similar to the mouth samples, the relative abundance of *Isotricha* decreased from 6.4% to 0 from day 30 to day 180 (*P* < 0.05, Fig. 5C). To better understand the temporal distribution of protozoa, we compared the dominant protozoal genera among these four sample sites across ages. *Dasytricha* was detected in the rumen of calves from day 120 but was detected in maternal samples at all age groups (Fig. 5C); whereas *Isotricha* was detected in the dams’ rumen, mouth, and skin samples but not in the rumen of calves (Fig. 5C). In addition, the dynamic relative abundance changes of *Entodinium* were similar among the calf’s rumen, and dam’s rumen and skin, namely, decreasing from day 30 to day 90, and then, increasing afterwards (*P* < 0.05, Fig. 5C).

### Trans-domain relationships between rumen fungi and protozoa of calves

**Fig. 6:**
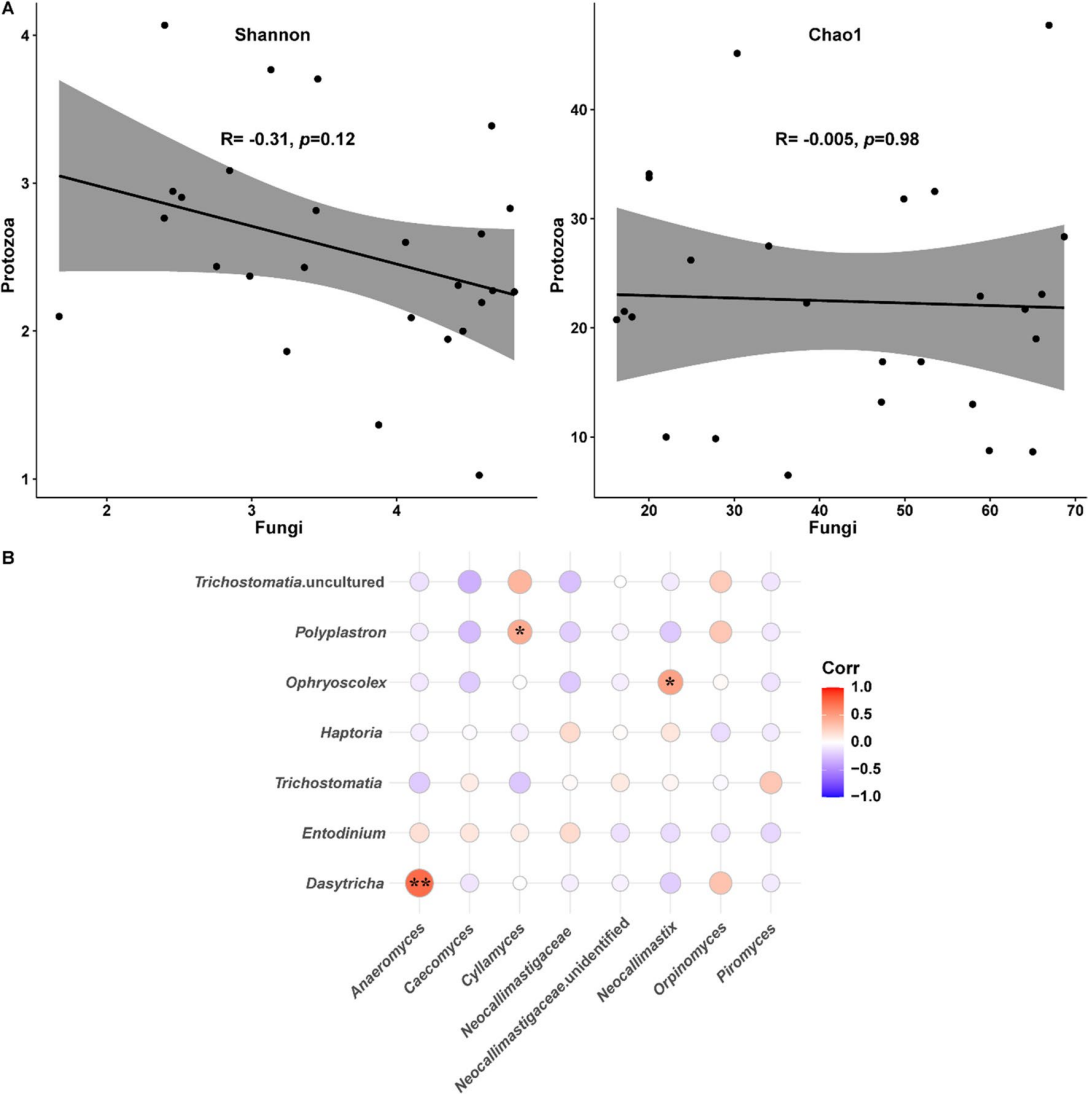
Trans-domain relationships between fungi and protozoa in the rumen of yak calves. **A** Correlations between fungi and protozoa alpha diversities by Shannon and Chao1 indices in the rumen of yak calves across ages. **B** Correlations between fungi and protozoa main genera in the rumen of yak calves. *P < 0.05, **P < 0.01. *x*-axis denotes the main fungal genera while the *y*-axis denotes the main protozoal genera

To gain an insight into the potential relationship between the developmental processes of rumen fungi and protozoa in calves, a Spearman correlation analysis, based on the alpha diversity indices and the relative abundances of the main eukaryotic microorganism at the genus level, was used. Fungal and protozoal diversity were correlated inversely (Fig. 6A), suggesting an overall antagonistic relationship between them in the rumen of calves. We further investigated the trans-domain interactions across fungal and protozoal genera according to their relative abundances in the rumen of calves across ages. A larger number of negative than positive correlations (34 vs. 22) emerged between fungi and protozoa (Fig. 6B). Among them, *Piromyces* correlated negatively with protozoal taxa, except for *Trichostomatia*, while *Cyllamyces* correlated positively with protozoal taxa, except for *Trichostomatia* and *Haptoria* (Fig. 6B). Notably, the fungal genera *Anaeromyces*, *Cyllamyces*, and *Neocallimastix* correlated positively with *Dasytricha*, *Polyplastron*, and *Ophryoscolex*, respectively (*P* < 0.05, Fig. 6B).

### Potential maternal sources for rumen fungi and protozoa in calves

**Fig. 7:**
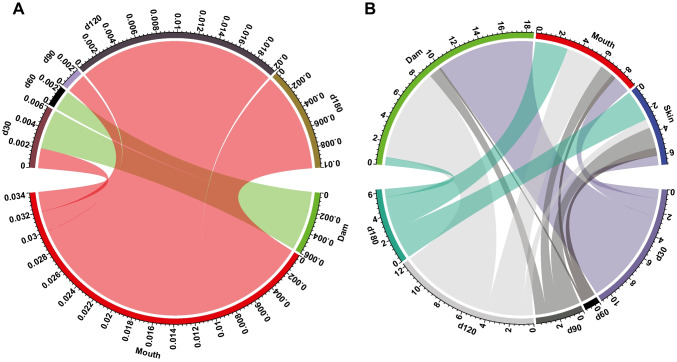
SourceTracker analysis of microbiota from different maternal sites in the rumen of calves. Proportion of rumen fungi (**A**) and protozoa (**B**) of yak calves from rumen, mouth, and teat skin (skin) of dams at different age groups. The number of outside circles means the proportion of contributions from different maternal sites to the rumen eukaryotes of calves, and different colors represent different maternal sites. The width of the end point of the band indicates the proportion of different maternal sites

SourceTracker analysis revealed that the mouth and rumen fluid of dams contributed to the development of rumen fungi in calves, with the mouth fungi contributing the greatest proportion to the calf rumen (Supplemental Fig. S2A). The contribution of rumen fungi of dams to rumen fungi in calves decreased with age of calves (*P* < 0.05, Fig. 7A). In contrast, mouth fungi accounted for a growing proportion of fungi in the calves’ rumen from day 30 to day 180, irrespective of a fluctuation at day 90 (*P* < 0.05, Fig. 7A). These findings indicate that the early fungal colonization in the rumen is affected by sampling sites. The rumen protozoa of dams contributed to 3.7% of calf’s rumen protozoa, while the mouth protozoa contributed 2.0% and skin protozoa contributed 1.4% (Supplemental Fig. S2B). The contributions of these three sites to rumen protozoa of the calves varied with age (Fig. 7B). For example, the contributions of protozoa from skin and mouth increased with the age of the calf except for some fluctuations at days 60 and 120 (*P* < 0.05, Fig. 7B; Supplemental Table S3), whereas the contribution from the rumen of dams fluctuated with the age of the calf (*P* < 0.05, Fig. 7B; Supplemental Table S3).

## Discussion

The neonatal rumen fungi and protozoa influence or even define the community of rumen fungi and protozoa in adulthood. It is thought that the assembly of these communities affect the health and production of the host in later life. However, how the neonatal rumen eukaryotes are populated initially remains unclear. Here, the transmission trajectory of fungi and protozoa from dams to yak calves and the interactions between rumen fungi and protozoa in yak calves were determined from day 30 to day 180 of age.

The alpha diversity of fungi in the rumen of yak calves decreased from days 90 to 180, which is consistent with previous studies in lambs and yak calves (Belanche et al. 2019; Guo et al. 2020a, b). This is likely due to a few taxa dominating the fungal community during rumen development (Puniya et al. 2015; Belanche et al. 2019). Furthermore, the alpha diversity of protozoa in the rumen of yak calves increased from day 60 to day 180, which supports the data reported by Guo et al. ([Bibr CR15][Bibr CR16]), where the protozoal alpha diversity in the rumen of yak calves increased from 2 months to 2 years of age. Rumen protozoa play an important role in carbohydrate degradation, and its diversity increased with the increased fiber content (Ishaq et al. 2017). In the current study, the diet of yak calves shifted gradually from milk to natural pasture with high fiber content. The increased protozoal diversity facilitated the utilization of available substrates, which provided nutrients for other microbes in the rumen. In addition, the alpha diversity of protozoa was much higher in the mouth of dams than the rumen of yak calves from day 30 to day 120. The mouth possesses a variety of microbes and serves as a transient site for them (Kaan and Kahharova 2000). However, rumen protozoa are highly sensitive to oxygen (Fonty et al. 1987), and only those that can adapt to the rumen environment could colonize it, resulting in a lower protozoal diversity in the calf’s rumen than the dam’s mouth. Notably, the rumen bacterial and archaeal alpha diversities of yak calves increased with age in our previous studies (Guo et al. 2022) on the same animals as in the current study. Rumen fungi and bacteria cooperate in the degradation of fibrous feed (Han et al. 2019), and rumen protozoa contain endo-symbiotic archaeal populations and provide the substrate (H_2_) for methanogens to reduce CO_2_ to CH_4_ (Huws et al. 2018). Thus, we reasoned that the increase in alpha diversity of protozoa may support the increase in archaea, while the decrease in fungi may be due to the increase of bacteria that compensate most of the fiber degrading works. Integrated understanding of the developmental trajectory and the role of cross-domain interactions between rumen prokaryotes and eukaryotes, as well as the multifaceted functions of them in the rumen of neonatal ruminants are needed to be taken into consideration in developing rumen microbiota modulation strategies to improve animal phenotypes in the future.

As in the present study, *Cyllamyces*, *Orpinomyces*, and *Neocallimastix* were the dominant fungi genera in the rumen of goats, sheep, cattle, and yaks (Wang et al. 2019; Langda et al. 2020). These genera display cellulolytic and xylanolytic activities (Puniya et al. 2015), indicating that they are important for grazing ruminants consuming high-fiber diets. Furthermore, it was reported that *JF423626* and *AL8* were the dominant fungal genera in the mouth of sheep and dairy cattle, respectively (Kittelmann et al. 2015; Tapio et al. 2016). This contrasts with the present study, where *Orpinomyces* and *Caecomyces* were the dominant mouth fungi, which may be attributed to the difference in dietary composition (Kittelmann et al. 2015). The relative abundance of *Orpinomyces*, a strong cellulose degrader (Palma-Hidalgo et al. 2021), increased with age in the rumen of calves in the current study, with the increase in fiber intake of the growing calves. The presence of this genus in the rumen of calves increases the crude fiber digestion and volatile fatty acid (VFA) production (Sehgal et al. 2008). In addition, *Orpinomyces* also plays an important role in ATP generation (Stairs et al. 2015), and therefore, its increased abundance as the calf ages would be beneficial for energy generation when the dietary intake is more fibrous and less digestible. Recent research revealed that *Dasytricha* dominated in the rumen of different ruminant species (Park et al. 2020; Bailoni et al. 2021; Palma-Hidalgo et al. 2021), which was supported in the current study. Moreover, the relative abundance of *Dasytricha* correlated positively with fiber digestion and VFA production (Mao et al. 2016; Palma-Hidalgo et al. 2021). In addition, *Dasytricha* exhibits glucosidase activity, and the glucose produced could be used for butyrate production (Yarlett et al. 1985), which stimulates the growth and development of rumen papillae (Park et al. 2022). Consequently, we reasoned that the changes in the relative abundance of *Dasytricha* reflects rumen development of grazing yak calves.

In line with previous studies, *Entodinium* was reported to be dominant in the mouth of sheep and dairy cattle (Kittelmann et al. 2015; Tapio et al. 2016). However, *Dasytricha* was prevalent in this study, but was detected at only very low abundance in the mouth of sheep and dairy cattle (Kittelmann et al. 2015; Tapio et al. 2016). This difference is likely due to the different diets and host species among studies (Dybicz et al. 2018). Future metagenome/metatranscriptome studies are warranted to explore the functions of protozoa in the mouth that might originate from the external environment, such as grass, soil, and feces, considering their importance to the host’s health (Deng et al. 2017).

Considering the importance of rumen fungi and protozoa in the digestion of fibers, we further examined the relationship between fungi and protozoa in the rumen of yak calves. Negative correlations between fungal and protozoal alpha diversity emerged. It was proposed that rumen protozoa predate rumen fungi and bacteria by producing relevant enzymes (Williams et al. 2020), and, thus, an overall antagonistic relationship between them could be expected. This was supported by the larger number of negative than positive correlations between fungal and protozoal genera in the rumen of yak calves. However, there were some important positive correlations between them. For example, *Anaeromyces* correlated positively with *Dasytricha*. *Anaeromyces* prefers glucose (Solomon et al. 2016), and *Dasytricha* exhibits glucosidase activity; thus, the prevalence of *Dasytricha* promotes the growth of *Anaeromyces*. *Neocallimastix* and *Ophryoscolex* correlated positively in the current study; both genera digest fibers with *Ophryoscolex* using cellulases (Rabee et al. 2019; Park et al. 2020). In addition, rumen protozoa contribute to carbohydrate breakdown (cellulose activity), and this function could be acquired via horizontal gene transfer from fungi (Williams et al. 2020). These findings indicate a fungal-protozoal mutualism in fiber digestion (Mizrahi and Jami 2018). Future studies are required to link these observations with the mechanisms of fiber digestion, such as enzymes or pathways associated with lignocellulose degradation. Altogether, these data underpin intricate trans-domain interactions between fungi and protozoa in the rumen of yak calves that may have joint functions in rumen microbiota development and maturation.

We identified taxa with high relative abundances that were shared by dams and calves. Some of these (e.g., *Orpinomyces*, f.*Trichostomatia*, and *Entodinium*) displayed similar dynamic patterns in relative abundance between dams and calves, confirming to a certain extent that dams are a potential reservoir of eukaryotes transmissible to calves. However, it remains uncertain whether the same species is transmitted to the calves from the dams or if an alternative transmission route is involved, although some taxa (e.g., *Orpinomyces*, *Polyplastron*, and *Dasytricha*) were prevalent in maternal samples across ages but appeared only at specific ages (days 120 and 180) in the rumen of calves. Besides, some taxa (e.g., *Anaeromyces*, *Oontomyces*, and *Isotricha*) prevailed in the maternal samples but were barely detectable in the rumen of calves. This suggests that early microbial colonization in the rumen is confined, rather than occurring by chance. The proportion of calves’ rumen eukaryotes in the maternal-sourced samples varied with sites, which suggests that the site affects the early microbial colonization in the rumen (Yeoman et al. 2018). Moreover, the dam’s rumen protozoa were the major source of the calf’s rumen protozoa, suggesting vertical transmission from the rumen community of dams to the newborn. However, only a minor proportion of the calves’ rumen fungi were derived from maternal-sourced fungi, indicating the rumen fungi are likely from other sources (e.g., vagina) or the inanimate environment (e.g., soil, water). Given the limitations of this study, further large-scale population studies based on metagenomics or metatranscriptomics sequencing are needed to distinguish which microorganisms originated from dams at a higher resolution level (species or strain). Furthermore, other sources of microbes, including soil, water, and grass, should be taken into consideration when exploring the origin of rumen fungi and protozoa of neonates since the inanimate environment is the most important source (Palma-Hidalgo et al. 2021). Nonetheless, this present study highlights that the acquisition of rumen fungi and protozoa by yak calves clearly differ.

In conclusion, the present study determined the contribution of different maternal sites, namely, rumen, mouth, and skin, to the acquisition and development of rumen fungi and protozoa in sucking and grazing yak calves between 30 and 180 days of age. The rumen fungi and protozoa of calves originated mainly from the dam rumen between days 30 and 60, then decreased afterward, indicating the importance of the rumen eukaryotes of dams to the colonization of rumen eukaryotes in calves in early life. The contribution of the mouth and skin to the rumen protozoa of calves increased with age, demonstrating the importance of these sites when the contribution of the dam’s rumen protozoa started decreasing. In addition, the divergence in inter-generation transmissibility between rumen fungi and protozoa indicates that the origins of these eukaryote differ.

## Supplementary Information

Below is the link to the electronic supplementary material.

Supplementary file1 (PDF 236 KB)

## Data Availability

The data generated during the current study are available from the corresponding author on reasonable request.

## Declarations

**Ethical approval**

All procedures on the yaks were approved by the Animal Ethics Committee of the Chinese Academy of Lanzhou University (permit number: SCXK Gan 20140215).
