# Supplementary material for: Transmission of fungi and protozoa under grazing conditions from lactating yaks to sucking yak calves in early life
Source: Appl Microbiol Biotechnol. 2023 Jun 21;107(15):4931–45. doi: 10.1007/s00253-023-12616-y (PMC10345012; doi:10.1007/s00253-023-12616-y)
Supplement: Supplementary file 1 — Supplementary file1 (PDF 236 KB) [file 253_2023_12616_MOESM1_ESM.pdf]

Supplementary material prepared for Applied Microbiology and Biotechnology

Transmission of fungi and protozoa under grazing conditions from lactating yaks to sucking yak calves in early life

Wei Guo[1,2,3], Tingmei Liu[1], André Luis Alves Neves[4], Ruijun Long[2], Allan Degen[5], Mi Zhou[3]*, Xiang Chen[1]*

[1] Key Laboratory of Animal Genetics, Breeding and Reproduction in the Plateau Mountainous Region, Ministry of Education, College of Animal Science, Guizhou University, Guiyang 550025, China

[2] State Key Laboratory of Grassland Agro-ecosystems, International Centre of Tibetan Plateau Ecosystem Management, School of Life Sciences, Lanzhou University, Lanzhou 730000, China

[3] Department of Agricultural, Food and Nutritional Science, University of Alberta, Edmonton, AB T6G 2P5 Alberta, Canada

[4] Department of Veterinary and Animal Sciences, Faculty of Health and Medical Sciences, University of Copenhagen, Grønnegårdsvej 3, DK-1870, Frederiksberg C, Denmark

[5] Desert Animal Adaptations and Husbandry, Wyler Department of Dryland Agriculture, Blaustein Institutes for Desert Research, Ben-Gurion University of the Negev, 8410500 Beer Sheva, Israel

* Corresponding author:

Mi Zhou: email: mzhou2@ualberta.ca

Xiang Chen: email: xchen2@gzu.edu.cn

Supplemental Table S1 The relative abundances of identified phyla among different sample sites at different age groups (%)

Microbial group	Age group	Phylum	Calf	Dam	Mouth	Skin	SEM
Fungi	d30	*Neocallimastigomycota*	100	99.9	99.9		0.0004
	d60	*Neocallimastigomycota*	100	100	99.4		0.002
	d90	*Neocallimastigomycota*	99.8	99.9	100		0.001
	d120	*Neocallimastigomycota*	99.8	100	100		0.001
	d180	*Neocallimastigomycota*	99.6	99.9	100		0.002
Protozoa	d30	*Archaeplastida*	0	0	0	1	0.003
		Opisthokonta	1.7	0	0.15	0	0.004
		SAR	95.4	100	98.8	94.5	0.01
		Unassigned	2.9	0	1	4.4	0.01
	d60	*Archaeplastida*	0	0	0	2.6	0.006
		Opisthokonta	0	0	0	0.1	0.0002
		SAR	99.4	99.9	99.5	90.3	0.02
		Unassigned	0.6	0	0.4	7	0.02
	d90	*Archaeplastida*	0	0	0.13	0	0.0002
		Opisthokonta	0.23	0	0.4	0	0.001
		SAR	99.6	99.8	95.6	99	0.01
		Unassigned	0.1	0.2	3.8	1	0.006
	d120	*Archaeplastida*	0	0	0.67	1.3	0.004
		Opisthokonta	0	0	1.1	0.6	0.003
		SAR	99.9	100	94.3	96.6	0.01
		Unassigned	0	0	3.9	1.5	0.007
	d180	*Archaeplastida*	0	0	0	0.3	0.0004
		Opisthokonta	0	0	0	0.6	0.001

	SAR	1	100	99.6	97	0.004
	Unassigned	0	0	0.3	2.1	0.003

Supplemental Table S2 The relative abundances of identified genera among different sample sites at different age groups (%)

Microbial group	Age group	Genera	Calf	Dam	Mouth	Skin	SEM
Fungi	d30	*Anaeromyces*	0	4.8	1.8		0.009
		Caecomyces	33.4	4.7	8.1		0.05
		Cyllamyces	1.7	1.8	10.9		0.02
		f.*Neocallimastigaceae*	36.8	19.5	40.3		0.06
		unidentified *Neocallimastigaceae*	0	0	0		0
		Neocallimastix	25.3	0	0		0.04
		Orpinomyces	1.9	68.1	35.2		0.08
		Piromyces	0	0	0		0
		Oontomyces	0	0	2.5		0.01
	d60	*Anaeromyces*	1.3	3.4	3.9		0.01
		Caecomyces	30.2	1.1	9.2		0.05
		Cyllamyces	0	0	7.9		0.01
		f.*Neocallimastigaceae*	27.9	18.3	28.3		0.05
		unidentified *Neocallimastigaceae*	19.3	0	0		0.06
		Neocallimastix	18.2	0	1.3		0.03
		Orpinomyces	0	76.5	32		0.09
		Piromyces	1.9	0	0		0.01
		Oontomyces	0	0	16.8		0.04
	d90	*Anaeromyces*	0	6.9	2.9		0.02
		Caecomyces	41.3	7.6	7.6		0.05
		Cyllamyces	0	0	0		0
		f.*Neocallimastigaceae*	23.7	8.6	3.6		0.04
		unidentified *Neocallimastigaceae*	0	0	0		0

		Neocallimastix	28.9	0	0		0.06
		Orpinomyces	0	76.4	78.1		0.1
		Piromyces	4.1	0	0		0.01
		Oontomyces	0	0	7.1		0.02
	d120	*Anaeromyces*	1.3	3.4	2.4		0.01
		Caecomyces	13.3	3.1	8.2		0.03
		Cyllamyces	0	0	1.5		0.004
		f.*Neocallimastigaceae*	26.6	7.1	16.2		0.04
		unidentified *Neocallimastigaceae*	0	0	0		0
		Neocallimastix	18.6	0	0		0.04
		Orpinomyces	37.2	85.8	65.9		0.08
		Piromyces	1.5	0	0		0.01
		Oontomyces	0	0	5		0.01
	d180	*Anaeromyces*	0	1.2	6.5		0.01
		Caecomyces	10.5	10.4	6.2		0.08
		Cyllamyces	37	1.2	2.7		0.09
		f.*Neocallimastigaceae*	6.5	14.8	8.6		0.02
		unidentified *Neocallimastigaceae*	0	0	1.9		0.004
		Neocallimastix	6.2	5.2	2.5		0.02
		Orpinomyces	37.8	65.5	11		0.1
		Piromyces	0	0	0		0
		Oontomyces	0	0	4.5		0.01
Protozoa	d30	*Dasytricha*	0	8	2.9	4.5	0.01
		Entodinium	58.5	14.7	33.6	24.6	0.06
		f.*Trichostomatia*	34.2	32.4	51.6	36.8	0.05
		Haptoria	1.1	0	0	0	0.003
		Ophryoscolex	0	0	0	0	0
		Polyplastron	0	2.1	1.2	12.4	0.03

	Trichostomatia;uncultured	1.2	15.2	1.5	0	0.02
	Isotricha	0	26.9	5.9	6.4	0.03
	Trichostomatia;Ambiguous_taxa	0	0	0	0	0
	Tracheophyta	0	0	0	1	0.003
d60	*Dasytricha*	0	4	6.4	0	0.01
	Entodinium	41.6	17.1	33.3	10.5	0.05
	f.*Trichostomatia*	56.8	73.9	45.9	62.9	0.07
	Haptoria	0	0	0	0	0
	Ophryoscolex	0	0	0	0	0
	Polyplastron	0	0	2.5	0	0.003
	Trichostomatia;uncultured	0	2.6	3.3	1.8	0.006
	Isotricha	0	1.4	6.7	0	0.008
	Trichostomatia;Ambiguous_taxa	0	0	0	0	0
	Tracheophyta	0	0	0	2.6	0.006
d90	*Dasytricha*	0	4.4	3.3	4.9	0.01
	Entodinium	5.7	3.4	14.4	13.4	0.02
	f.*Trichostomatia*	93.2	89	69.3	57.2	0.05
	Haptoria	0	0	0	0	0
	Ophryoscolex	0	0	0	0	0
	Polyplastron	0	1	1.2	0	0.002
	Trichostomatia;uncultured	0	1.4	2.2	3.4	0.006
	Isotricha	0	0	3.7	0	0.005
	Trichostomatia;Ambiguous_taxa	0	0	1	0	0.001
	Tracheophyta	0	0	0	0	0
d120	*Dasytricha*	5.5	0.01	0.03	13.4	0.02
	Entodinium	21.8	8.5	28.6	7.8	0.03
	f.*Trichostomatia*	52.5	85.2	47.2	64	0.05
	Haptoria	0	0	0	0	0

	Ophryoscolex	12.3	0	1.1	1.4	0.02
	Polyplastron	0	0	4.4	0	0.01
	Trichostomatia;uncultured	3.9	4.5	8	0	0.01
	Isotricha	0	0	0	0	0
	Trichostomatia;Ambiguous_taxa	0	0	0	0	0
	Tracheophyta	0	0	0	0	0
d180	*Dasytricha*	20.8	10.8	5.1	6.2	0.03
	Entodinium	9.5	14.3	37.1	16.4	0.03
	f.*Trichostomatia*	16.4	48.1	50.3	50	0.06
	Haptoria	0	0	0	0	0
	Ophryoscolex	6.5	1.2	1.7	3.9	0.01
	Polyplastron	16.6	8.4	1	1.1	0.02
	Trichostomatia;uncultured	30.1	10.7	2.5	17.4	0.04
	Isotricha	0	4.3	1.2	0	0.01
	Trichostomatia;Ambiguous_taxa	0	1.3	0	0	0.002
	Tracheophyta	0	0	0	0	0

Supplemental Table S3 Proportion of rumen protozoa in yak calves originated from different maternal sites at different age groups

Source	Age					SEM	*P*-value
	d30	d60	d90	d120	d180		
Dam	8.53	0.17	1	8.17	0.71	0.03	0.02
Mouth	1.54	0.42	1.17	3.32	2.86	0.01	0.01
Skin	0.85	0.7	1.82	0.78	2.75	0.002	0.003

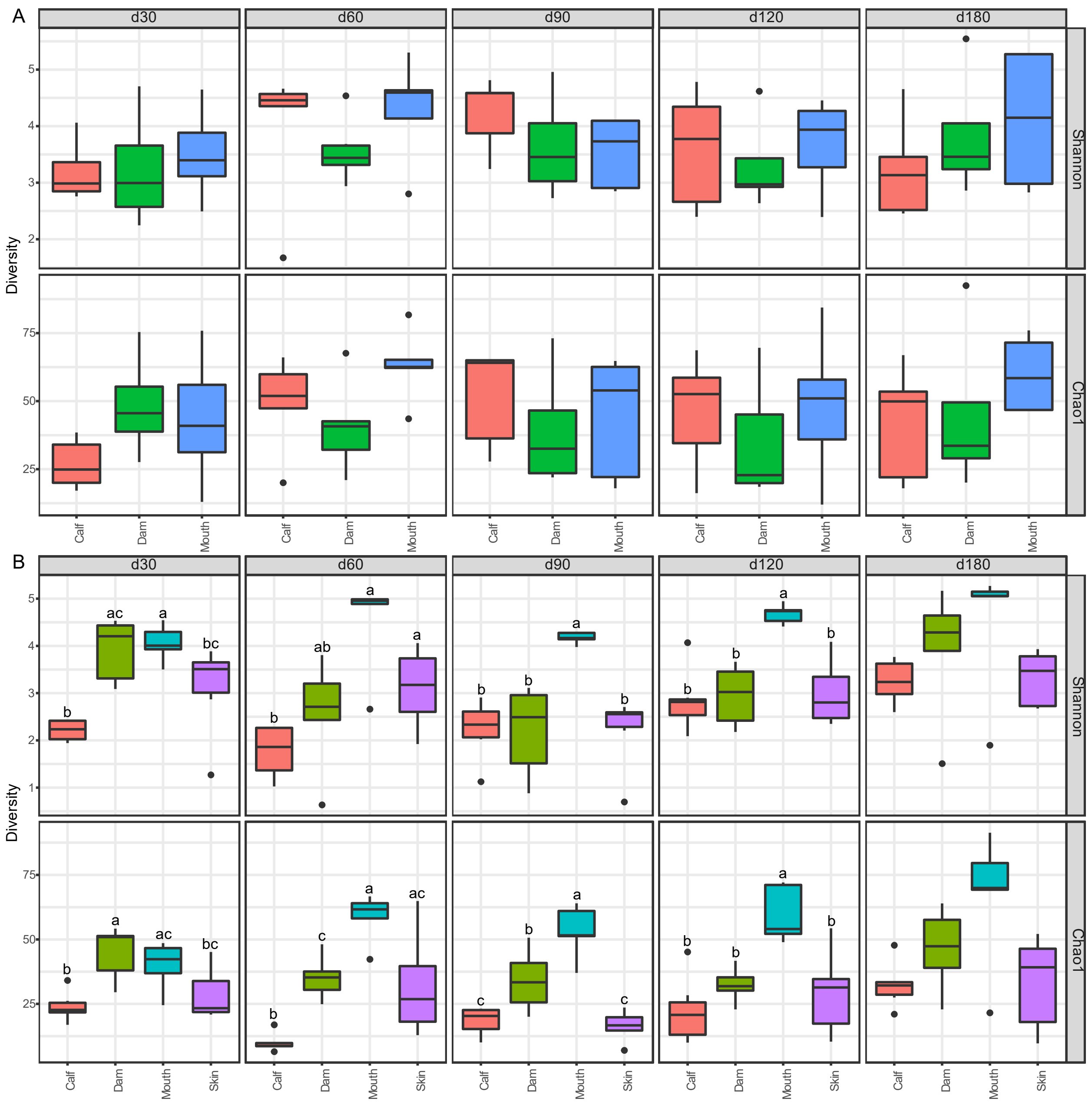

Supplemental Fig. S1 Alpha diversity of fungi (A) and protozoa (B) at different sites and different ages of yak calves. Statistical significance was determined using Non-parametric Kruskal-Wallis and Duncan's tests among sites within each age group. A p-value < 0.05 was accepted as significant. Means within an age group followed by different lowercase superscripts differ from each other.

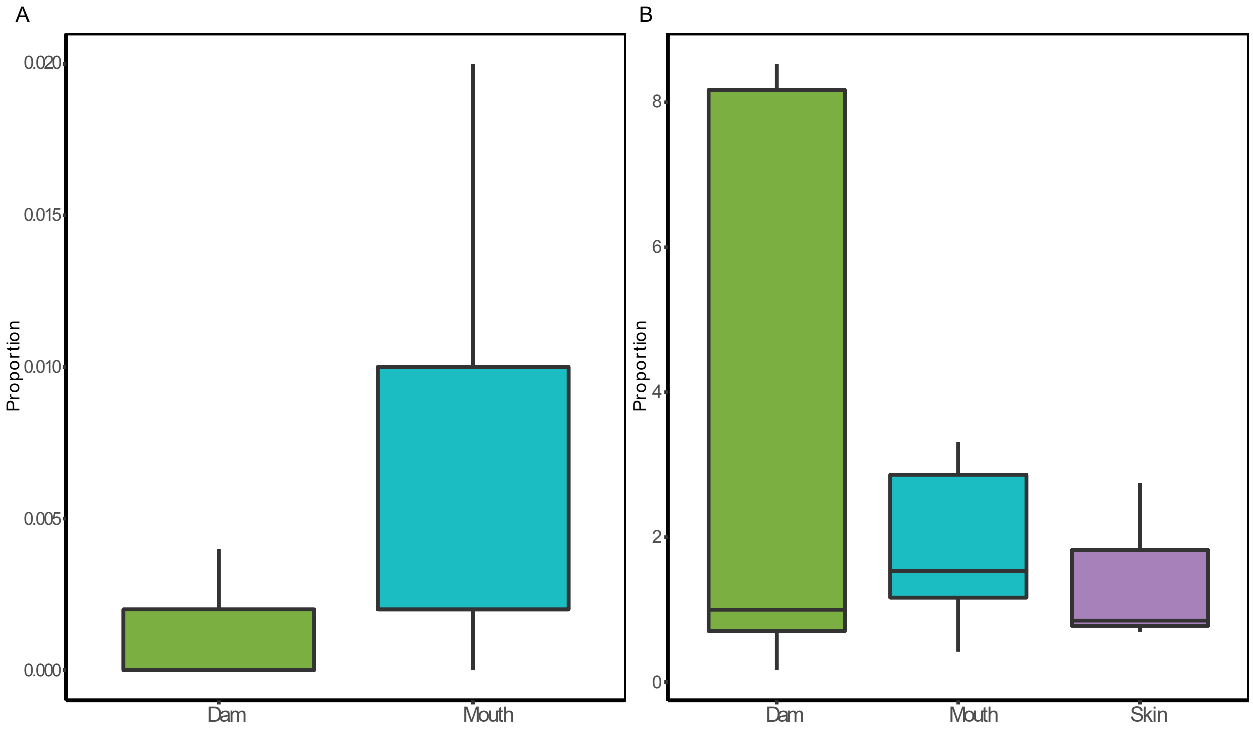

Supplemental Fig. S2 Source Tracker analysis of microbiota from different maternal sites in the rumen of calves. Proportion of rumen fungi (A) and protozoa (B) of yak calves from rumen, mouth, and teat skin of dams.
